# Biochar for the Removal of Emerging Pollutants from Aquatic Systems: A Review

**DOI:** 10.3390/ijerph20031679

**Published:** 2023-01-17

**Authors:** Mingying Dong, Lizhi He, Mengyuan Jiang, Yi Zhu, Jie Wang, Williamson Gustave, Shuo Wang, Yun Deng, Xiaokai Zhang, Zhenyu Wang

**Affiliations:** 1 Institute of Environmental Processes and Pollution Control, School of Environmental and Civil Engineering, Jiangnan University, Wuxi 214122, China; 2 Key Laboratory of Soil Contamination Bioremediation of Zhejiang Province, Zhejiang A & F University, Lin’an 311300, China; 3 School of Chemistry, Environmental & Life Sciences, University of the Bahamas, Nassau 4912, Bahamas; 4 Jiangsu Collaborative Innovation Center of Technology and Material of Water Treatment, Suzhou University of Science and Technology, Suzhou 215009, China

**Keywords:** biochar, microplastics, organic pollutants, endocrine disrupting chemicals, pharmaceutical and personal care products, water pollution

## Abstract

Water contaminated with emerging pollutants has become a serious environmental issue globally. Biochar is a porous and carbon-rich material produced from biomass pyrolysis and has the potential to be used as an integrated adsorptive material. Many studies have shown that biochar is capable to adsorb emerging pollutants from aquatic systems and could be used to solve the water pollution problem. Here, we provided a dual perspective on removing emerging pollutants from aquatic systems using biochar and analyzed the emerging pollutant removal efficiency from the aspects of biochar types, pollutant types and coexistence with heavy metals, as well as the associated mechanisms. The potential risks and future research directions of biochar utilization are also presented. This review aims to assist researchers interested in using biochar for emerging pollutants remediation in aquatic systems and facilitate research on emerging pollutants removal, thereby reducing their environmental risk.

## 1. Introduction

**Figure 1:**
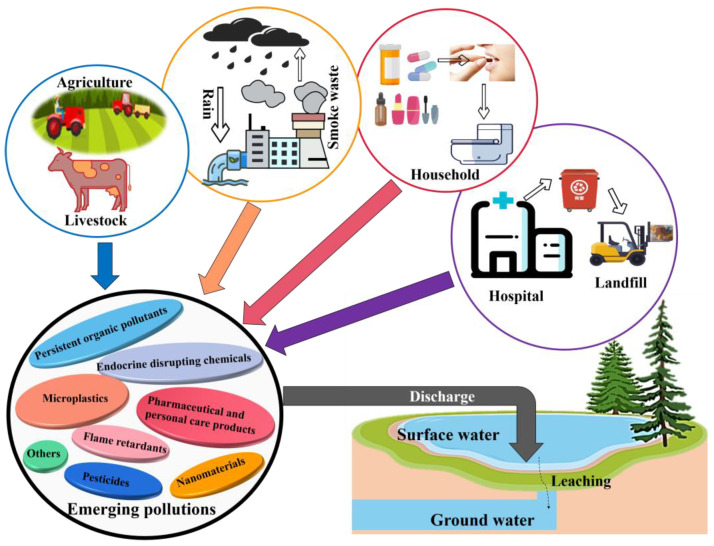
Resource of emerging pollutants and routes to the aquatic system.

Rapid industrialization, urbanization and the excessive use of agrochemicals resulted in a significant reduction in surface water quality [1,2]. Thus, water pollution is now a serious environmental issue for humans [3]; emerging pollutants are the most common and non-negligible due to lack of monitoring [4,5,6]. Emerging pollutants commonly originate from the release of hospital and factory wastewater, residential sewage and agricultural runoff (see Figure 1) and include endocrine-disrupting chemicals (EDCs), microplastics (MPs), pesticides, flame retardants, nanomaterials, pharmaceutical and personal care products (PPCPs). The pollution poses a serious threat to drinking water quality. Some emerging pollutants are persistent organic pollutants that are not easily degraded and have a long-term presence in the aquatic environment, presenting a “pseudo-persistent existence” state and causing many adverse effects on the environment [4]. The treatment of these pollutants has increasingly become a big challenge in recent years [7]. Therefore, it is imperative to develop proper methods to treat emerging pollutants in water [8] for the benefit of the environment, public health and economic activities.

Many chemical, physical and biological methods, such as membrane filtration, coagulation, adsorption, photocatalytic degradation, aerobic bioreactors and activated sludge have been used to remediate polluted waters [9]. The type of process that is involved in each method usually depends on the pollutant nature and the environmental conditions [10]. In recent decades, biochar, which is a carbon-rich material derived from the pyrolysis of biomass [11,12,13], has been shown to be efficient in adsorbing pollutants of wastewater [14,15]. The application of biochar in the environmental field has also been a hot research topic in recent years. This is mainly due to biochar as an adsorbent, is an environmentally friendly material and has excellent removal efficiency for pollutants. Thus, the purpose of this review article is to deliver a balanced view of the current practices on various aspects of biochar utilization for emerging pollutants removal from aquatic systems. In this paper, we reviewed the latest studies on the application of biochar for the remediation of different emerging pollutants in aquatic systems. Factors affecting the adsorption performance of biochar and the adsorption mechanisms of different types of emerging contaminants were summarized. Finally, future research directions associated with biochar use in polluted water remediation were discussed.

## 2. Removal of Emerging Pollutants by Biochar

It has been reported that wastewater containing amounts of emerging pollutants displayed detrimental effects on human and ecosystem health [16]. For example, EDCs, a major component of medical wastewater, are suspected to be harmful to humans and wildlife. Previous studies demonstrated that MPs and PPCPs can directly or indirectly cause risk to the ecosystem and people’s health [17,18]. It is crucial to remove these pollutants from aquatic ecosystems. In this paper, we have reviewed the studies on the removal of three typical emerging pollutants by biochar. Numerous investigations have been conducted to study biochar’s adsorptive capacity for these emerging pollutants in aquatic environments [3,5,6] and these studies have been discussed in the following sections.

### 2.1. Removal of Endocrine Disrupting Chemicals (EDCs)

EDCs are a subclass of compounds that mimic the actions of hormones and disturb the endocrine system’s normal functions, resulting in malignant tumors, birth deformities and developmental disorders [19]. Previous studies have revealed that biochar can remove EDCs from aquatic systems and significantly reduce their environmental risk. The sorption of EDCs by biochar is largely determined by its specific surface area and the functional groups which are strongly affected by the raw material and the pyrolysis temperature [20,21]. In addition, raw agricultural by-products without pyrolysis might leach pollutants and contribute to secondary pollution, but pyrolysis to biochar can completely decompose most organic pollutants and avoid this problem [22,23]. As shown in Table 1, biochar produced from different feedstocks and pyrolysis temperatures usually had a high adsorptive potential for EDCs. For example, Sun et al. [24] reported that peanut shell biochar had a strong adsorption capacity of all six endocrine-disrupting phenols which can be attributed to the abundant aromatic carbon, aliphatic structures and functional groups of biochar surface.

The temperature at which biochar is pyrolyzed will significantly alter its characteristics, thereby affecting its adsorption capacity on the pollutants. Generally, high pyrolysis temperature will decrease the biochar surface functional groups and increase its aromaticity and specific surface area. For example, biochar obtained at low temperatures (e.g., lower than 400 °C) will form more functional groups and have stronger adsorptive capacities for pollutants [25]. Other research found that higher-temperature biochar had a significantly higher adsorption capacity for diethyl phthalate (DEP) than lower-temperature biochar, which reflected the increased aromaticity and probable dominance of the π-π electron donor-acceptor (EDA) interaction [26]. Sun et al. [27] investigated the ability of different biochars to absorb three phthalic acid esters (PAEs) in water. They reported that the adsorption follows an order: torrified plant material biochar (200 °C) < turbostratic biochar (600–700 °C) = composite biochar (500–600 °C) < transition biochar (300 °C) < amorphous biochar (400 °C). This most likely occurred because low-temperature biochars have a more amorphous structure and include significant amounts of “soft” aliphatic domains, making them an efficient medium for hydrophobic organic molecules partitioning [28].

**Table 1:** Representative studies on the removal of endocrine-disrupting chemicals from aquatic systems by biochar.

Feedstocks	Production Temperature	Pollutants	Results	References
Eucalyptus globulus	400 °C and 600 °C	Estrone (E1), 17β-estradiol (E2), estriol (E3), 17α-ethynylestradiol (EE2), bisphenol A (BPA) and 4-tert-butylphenol	The sorption capacities of biochar for different chemicals followed the order E1 > E2 ≥ EE2 > BPA > 4tBP > E3.	[29]
Spent mushroom substrate	250 °C, 450 °C and 600 °C	EE2 and progesterone	The addition of biochar removed 80% of both endocrine disruptors.	[30]
Grapefruit peel	400 °C	BPA	Biochar strongly enhanced the removal rate of BPA through adsorption.	[31]
Red algae	300–900 °C	4-Nonylphenol	Algal-derived biochar can be a sustainable material for the decomposition of 4-Nonylphenol.	[32]
Peanut shells	400 °C	BPA, diethylstilbestrol, hexafluorobisphenol, 4,4-sulfonyldiphenol, 4,4-methylenebisphenol and 4-cumylphenol	Biochar is capable to remove EDCs from aqueous.	[24]
Oil palm fibre	200 °C, 350 °C, 500 °C and 700 °C	Ethylparaben	Ethylparaben was able to bind to biochar at an adsorption capacity of 349.65 mg/g.	[33]
Walnut shell	400 °C, 500 °C, 600 °C and 700 °C	Estrone	700 °C-Biochar at pH 4 with a dosage of 0.1 mg/mL showed the maximum surface assimilation of estrogens.	[34]
Sawdust	500 °C	E2	The result indicated that graphene-like magnetic biochar had the highest removal rate of E2.	[35]
Bagasse	400 °C, 600 °C and 800 °C	E2	Magnetic biochar nanoparticles can strongly adsorb E2 which makes them potential adsorbents for E2 removal.	[36]
Corn straw	700 °C	Perfluorooctane sulfonate (PFOS) and EE2	The concentration of PFOS significantly reduced after the addition of biochar.	[37]
Rice husk	300 °C	Propylparaben (PP)	The addition of biochar greatly promoted propylparaben degradation.	[38]

### 2.2. Removal of Microplastics (MPs)

An alarming increase in plastics in the aquatic environment has gradually raised scientists’ concerns about their potential risk to the environment in recent years [37]. Plastic waste can slowly degrade to a large number of small plastic pieces (MPs) as a result of biogeochemical processes [38]. MPs contamination in aquatic systems is a developing problem worldwide [39]. The additives in plastics (e.g., PAEs, flame retardants, alkyl phenol, azodicarbonamide) may enter the aquatic environment through aging processes and threaten aquatic ecosystems. Similarly, these MPs may threaten human health by entering the human body through the food chain. Furthermore, MPs can serve as a sink for different types of pollutants (e.g., PCBs, nonylphenol, and metals) [40,41] in the environment and further enhance the toxicity of MPs. Biochar has a high surface area and micropores that can adsorb MPs in water (see representative studies summarized in Table 2). For example, Ganie et al. [42] used bagasse biochar pyrolysis at 350, 550 and 750 °C to remove MPs from aquatic systems; 750 °C biochar had the highest MPs removal rate due to its porous structure and high surface area. Wang et al. [43] also reported that biochar produced at high temperatures displayed an enhanced removal and immobilization capacity for MPs (>95%). They further demonstrated that the presence of abundant honeycomb structures in thin chips prepared at 500 °C biochar contributed to their high adsorption capacity for MPs. Wang et al. [44] showed that magnetic biochar can remove up to 94.81% of the polystyrene (PS) microspheres from an aqueous solution. Iron-modified biochar has magnetic extractability to easily and quickly remove MPs [45]. Similarly, oxidized corncob biochar showed a relatively higher removal rate of polystyrene MPs (>90%) than normal biochar due to it having more hydroxyl groups [46].

In addition, environmental conditions will also affect biochar performance for MPs removal. Kumar et al. [47] reported that higher pH, dissolved organic matter and nutrient content may reduce biochar adsorption capabilities for MPs; however, an increase in the aquatic system’s temperature promotes MPs adsorption by biochar.

**Table 2:** Representative research on biochar’s ability to remove microplastics from aquatic environments.

Feedstocks	Production Temperature	Pollutants	Results	References
Cellulose	400 °C	Microplastics (MPs)	Biochar reduced the transit and increased the deposition of plastic particles.	[48]
Livestock manure	500 °C	Polyhydroxyalkanoate microplastics (PHA-MPs)	Biochar accelerated PHA-MPs biodegradation (degradation rate of 22–31%).	[49]
*Prosopis juliflora*	550 °C and 850 °C	MPs	Both biochars produced from the two temperatures can effectively remove the MPs (>200 mg/g).	[45]
Pine and spruce bark	475 °C and 800 °C	Spherical polyethylene (PE)	The steam-activated biochar was an excellent adsorbent for removing MPs.	[50]
Sawdust	550 °C	Polystyrene	Modified biochar showed high MPs removal efficiency (>94.8%).	[51]
Cellulose	400 °C	Polystyrene	Transport of plastic particles hindered by biochar added.	[52]
Sugarcane bagasse	350 °C, 550 °C and 750 °C	Polystyrene-based latex	Biochar prepared at 750 °C showed a higher MPs removal rate (>99%) compared with the lower temperature biochar.	[43]
Corn straw, hardwood	300 °C, 400 °C and 500 °C	MPs	Both 500 °C corn straw and hardwood biochar had higher removal and immobilization capacity of MPs than low-temperature biochar.	[42]

### 2.3. Removal of Pharmaceutical and Personal Care Products (PPCPs)

PPCPs may release harmful chemicals into aquatic systems and have adverse effects on aquatic life due to their widespread use and persistence when they are improperly disposed of [53]. Generally, PPCPs in water mainly originate from sewage treatment plants and landfills. Detectable concentrations can be found in drinking waters around the world and may pose a potential risk to ecosystems [54]. Although the long-term effects of PPCPs on living organisms are not clear [55], the removal and reduction of PPCPs are still required due to their potential risk. Biochar as a sorbent has been shown to be effective in removing PPCPs from aquatic environments (Table 3) [54]. For example, Liang et al. [56] reported 700 °C biochar exhibited a high adsorption capability for norfoxacin (NOR). It has also been shown that the modification of biochar can improve its adsorption capacity for PPCPs. Ahmad et al. [57] found that nano zero-valent iron-modified biochar (nZVI-DBC) could effectively remove 98% of chlortetracycline from aqueous solutions, which was significantly higher than zeolite-modified biochar (Z-DBC), silica modified biochar (S-DBC) and non-modified biochar. This is due to that after the oxidation of Fe^0^, the chlortetracycline’s amino group may have created Fe-N covalent connections with Fe^3+^ and Fe^2+^. In addition, after ball milling, hickory chip biochar (pyrolyzed at 700 °C) significantly adsorbs more emerging pollutants from water [32].

Some PPCPs can be removed through the specific properties of biochar. For example, the calcium present in the biochar produced from milk sludge can be used as a complexing precipitant for fluoride adsorption and removal [58]. In addition, PPCPs removal can also be enhanced via microbial degradation catalyzed by microbes colonizing the surface of biochar. The microbial activities are enhanced since biochar may serve as both an electron donor and acceptor for PPCPs mineralization [59]. According to the discussion above, biochar and modified biochar can effectively remove PPCPs from aquatic systems which may reduce the environmental risk of these emerging pollutants.

The adsorption capacities for various adsorbents tested for different emerging pollutants removal are summarized in Table 1, Table 2 and Table 3. However, due to the inconsistency of data in the literature, it is difficult to provide a direct comparison of the adsorption capacities of different biochars on any particular pollutant or pollutant groups. The adsorption capacities of different biochars for a range of pollutants have been reported in the literature under varied conditions (different solution pH, pyrolysis temperatures, pollutant concentrations, biochar types, as well as the biochar addition doses). Additionally, the biochars used in the adsorption studies under controlled laboratory settings with aquatic systems had the purpose of obtaining datasets to simulate the conditions under realistic field situations such as industrial wastewater, mining wastewater, and agricultural wastewater. No two experiments that were conducted by the different authors had a similar experimental protocol. Furthermore, the isotherm models used to derive the sorption parameters also varied among the studies and the findings often do not match thus making the comparison unrealistic.

**Table 3:** Representative studies on the removal of PPCPs from aquatic systems by biochar.

Feedstocks	Production Temperature	Pollutants	Results	References
Straw	300 °C and 600 °C	Sulfamethazine (SMT)	SMT sorption to biochar at 300 °C and 600 °C display their highest concentrations of 5.75 mg/g and 4.32 mg/g, respectively.	[60]
Poplar wood chips	700 °C, 800 °C and 900 °C	Norfoxacin (NOR)	The obtained 700 °C biochar exhibited a superior NOR adsorption capability (up to 38.77 mg/g).	[61]
Bagasse, bamboo, hickory chips	300 °C, 450 °C and 600 °C	Sulfamethoxazole (SMX), sulfapyridine (SPY)	The highest amounts of SMX and SPY that biochar could adsorb were 25.7 mg/g and 58.6 mg/g, respectively.	[32]
Cassava waste residues	500 °C	NOR, sulfamerazine (SMR), oxytetracycline (OTC)	Mono- and competitive sorption of three antibiotics to raw and NH^4+^- modified cassava biochar followed a similar order: OTC > NOR > SMR.	[62]
Bagasse	500 °C	SMX, thiazole, methylpyrimidine, dimethylpyrimidine	Great adsorption performance was demonstrated in the adsorption process of the four sulfonamide antibiotics under ideal circumstances, pH 4 and 35 °C.	[63]
Bamboo	500°C	Enrofloxacin andofloxacin	When the initial concentration of enrofloxacin or ofloxacin was increased from 1 to 200 mg/L, the adsorption capacity of bamboo biochar increased sharply and then began to flatten out with a further increase in the initial concentration.	[64]
Palm fruit empty bunch	250 °C, 450 °C and 750 °C	Methyl paraben (MPB), carbamazepine (CZP), ibuprofen (IBP), and triclosan (TCS)	450 °C-Biochar can remove more than 75% of the three organic pollutants.	[53]
Sewage sludge	600 °C	Tetracycline (TC), SMX, amoxicillin (AMC)	Biochar could adsorb TC, SMX, and AMC to maximum levels of 123.35, 99.01, and 109.89 mg/g, respectively.	[65]
Cornstalk, orange peel, peanut hull	300 °C, 500 °C and 700 °C	TC	The three different biochars’ ability to adsorb TC was greatly improved by the KMnO_4_ treatment.	[66]
Douglas fifer	900 °C and 600 °C	Fluoride	Both nitrate and fluoride adsorption on biochar remained high over a pH range from 2 to 10.	[67]
Food waste	300 °C, 450 °C and 600 °C	Fluoride	Excellent adsorption ability (91.4% removal) has been shown by the aluminum-modified biochar in the pH range of 5–11.	[68]

## 3. Factors Affect the Performance of Biochar for Emerging Pollutants Removal

### 3.1. Types of Biochar

Biochar with diverse properties may result in different performances when used for the remediation of contaminated water. It has been reported that biochar properties may be affected by preparation processes, raw material types, modification and aging methods, which lead to different surface functional groups, pH and specific surface area [51,69,70].

It was observed that the specific surface area and pore volume of coconut shell biochar obtained by supercritical water gasification were increased by 78 times and 43 times, respectively, as compared to those obtained by pyrolysis [51]. In addition, high temperatures may decrease biochar’s polarity and hydrophilicity, while the aromaticity and specific surface area gradually increased [71]. Moreover, the temperature during production can also alter biochar pH. For instance, the pH of four different kinds of biochar increased and the pH increase rate gradually slowed down with the increase in pyrolysis temperature [72]. This may be due to the decomposition of cellulose, hemicellulose and lignin in the raw material. Furthermore, the release of volatile substances resulted in pore structure opening and the increase in the specific surface area, which can significantly improve the biochar adsorption capability for organic pollutants [73]. Additionally, the retention time of pyrolysis significantly affects the biochar surface area which further affected the ability of biochar to adsorb phenol [74].

Some environmental factors, i.e., “aging” may also change the properties and structure of biochar. For example, rainfall or freezing and thawing will lead to mechanical cracking, surface oxidation, dissolved organic matter release and mineral dissolution of biochar [74,75,76,77]. Previous research showed that the surface oxidation of biochar through the aging process would increase nutrient mineralization and soil microbial activities, reducing the unstable biochar content [74,78,79]. However, the aging process would increase the acidic functional groups of biochar and reduce its adsorption performance [80]. In addition, long-term aging processes promote the polarity and hydrophilicity of biochar, which may increase the bioavailability of PAHs [81]. Zhang et al. [82] reported that aging significantly reduces the adsorption capacity of biochar for PAEs, mainly due to the clogging of its pores by dissolved organic carbon in the environment.

Modified biochar has gradually attracted researchers’ attention which is due to its higher adsorption capacity for pollutants compared to normal biochar [64,83]. For example, manganese dioxide-modified biochar (Mn-BC) obtained higher specific surface area, total pore volume and pore diameter and showed higher sorption capacity of organic pollutants compared to the original biochar [84]. Modification methods significantly affect the surface functional groups of biochar, which further affects the adsorption capacity of biochar to the targeted pollutants. It has been observed that MnCeOx-modified tea waste biochar (MnCeOx/TBC) not only improved the agglomeration of bimetallic oxides but also provided surface functional groups, which could synergistically adsorb pollutants in water and promote tetracycline surface oxidation [85]. When the biochar is modified by pretreating feedstock with sulfamic acid, it can be used as an electron donor and combine with an electron acceptor-TC molecule conjugated ketene structure through a π-π interaction [86].

### 3.2. Types of Emerging Pollutants

The type of emerging pollutants or their coexistence with other pollutants may be another factor affecting the removal capacity of biochar. The properties of organic pollutants, including molecular structure, hydrophobicity, aromaticity, and polarity, are the key factors affecting biochar removal capacity [87,88,89]. For example, pollutants with large kinetic diameters can be rapidly adsorbed into large pores and be easily blocked out by micropores and nanopores [90,91]. This results in different mobility between the different pollutants and biochar, which leads to differences in adsorption performance [92]. In addition, pollutants with higher hydrophobicity can be more easily removed by biochar, due to the better affinity of pollutants with hydrophobic biochar [93,94]. Besides, the π-π EDA interaction between the sorbent and biochar can be dominated by the aromaticity of organic pollutants, which may directly affect biochar removal capacity for pollutants [95]. The aromaticity of TC is higher than sulfadiazine; therefore, TC has more pronounced π-π interactions and higher adsorption affinity for biochar [96,97]. The polarity of aromatic rings on organic pollutants is closely related to the adsorption of biochar. The π-π interaction tends to be strongest between aromatic hydrocarbons with opposite polarity and weakest between aromatic hydrocarbons with the same polarity, which indicates that the polarization direction of different organic pollutants significantly affects the adsorption process [95]. However, the potential effect of polarization on the removal of organic pollutants by biochar has not been fully investigated.

However, the pollutants in the actual environment are often complex and diverse. The biochar adsorption capacities of specific pollutants may be various under the synergistic or competitive effect of multiple organic pollutants. For example, Luo et al. [62] found that biochar exhibited stronger adsorption capacity for NOR and oxytetracycline (OTC) but weaker adsorption capacity for sulfamerazine (SMR) in ternary solutions compared to single solutions, which was attributed to the electrostatic repulsion between the antibiotics in the accelerated adsorption phase. When the adsorption sites on biochar are occupied by antibiotics, these antibiotics adsorb more ions through electrostatic interaction of opposite charges, while electrostatic interactions inhibit the ability of SMR to compete for the adsorption sites on biochar [60]. However, in complex multicomponent systems, synergistic and competitive interactions of the different solutes do not necessarily exist simultaneously. It has been found that under competitive conditions where TC, sulfadiazine, NOR, erythromycin, and chloramphenicol were present simultaneously, all antibiotics exhibited competitive adsorption, which reduced the adsorption of biochar on individual pollutants [98]. The limited external and internal pores and spatial site resistance near the micropore adsorption domains are responsible for the weak adsorption affinity of the multi-solute system [99].

### 3.3. pH

The pH of the solution largely controls the adsorption effect of biochar on emerging pollutants. It has been reported that pH changes alter the physicochemical behavior of the functional groups on the surface of biochar [100]. When the solution pH is lower than the zero point charge (PZC), the functional groups on the surface of biochar protonate to obtain H^+^, which will compete with the positive organic pollutants on the surface for adsorption thus weakening the adsorption capacity of biochar [100]. However, the protonation of functional groups facilitates the adsorption of negative pollutants [101,102]. On the contrary, when the solution pH is higher than PZC, the loss of protonation occurs on the surface of biochar to reopen the adsorption sites occupied by H^+^, thus improving the adsorption of organic pollutants [103]. Taking TC as an example, under low pH conditions, positively charged contaminants, such as TCH_3_^+^, compete with H^+^ on the surface of biochar thereby reducing the amount of contaminants adsorbed by biochar [100]. However, under high pH conditions, negatively charged contaminants (e.g., TCH^−^ and TC_2_^−^) showed a lower affinity for the negatively charged biochar surface due to electrostatic repulsion [104].

### 3.4. Initial Emerging Pollutant Concentration

The initial concentration of emerging pollutants is one of the important parameters affecting the adsorption performance of biochar. At low initial concentrations, biochar can rapidly adsorb pollutants through adsorption sites because there are enough active sites to achieve low levels of emerging pollutant removal, which promotes high removal efficiency [105]. As the initial concentration increases, the removal efficiency of biochar gradually plateaus until saturation [31,50,106,107]. For instance, as the concentration of ofloxacin increased from 1 g/L to 4 g/L, the adsorption capacity of biochar decreased from 11 mg/L to 4 mg/L. As the initial concentration continued to increase to 5 g/L and 6 g/L, the adsorption capacity of biochar remained constant which is due to the transfer of emerging contaminants being limited by the low availability of binding sites on biochar at higher concentrations [108]. However, for the removal of emerging contaminants at high concentrations, an increasing dose of biochar can be considered [109], which will be discussed in the section below.

### 3.5. Biochar Dose

Generally, at high pollutant concentration conditions, the increase in the dosage of biochar can significantly improve the removal of emerging pollutants [110]. This is due to the high dosage of biochar providing more binding sites and a larger specific surface area, which allowed more contaminant molecules to be immobilized [48,111,112]. However, biochar above a certain concentration may cause an agglomeration effect, which reduces the total specific surface area of the biochar and inhibits the exposure of binding sites, so that the adsorption capacity of the biochar is weakened [113]. It has been reported that when the amount of biochar was increased from 2 g/L to 20 g/L, the adsorption of diclofenac by biochar decreased from 107% to 25% [114]. However, the agglomeration capacity of biochar was more dependent on the raw material. Biochar prepared from raw materials with high lignin content was more prone to agglomeration than biochar produced from raw materials with low lignin content [115]. This may be due to the inherent high adhesive capacity of lignin and the incomplete decomposition by pyrolysis [116]. Therefore, the optimal biochar dosage is an important parameter to ensure the adsorption efficiency of biochar as well as the cost-effectiveness of its use [83,117,118].

### 3.6. Coexistence of Heavy Metals and Emerging Pollutants

Wastewater from electroplating, leather tanning, textile and dyeing industries contains heavy metals and organic pollutants [119]. Due to unique physicochemical properties, biochar can adsorb pollutants from water systems, but the adsorption mechanism of organic pollutants is not the same as that of heavy metals, and the remediation effect of biochar varied when they coexisted [120,121]. Heavy metals and organic pollutants may compete for the adsorption sites of biochar in the aqueous phase [122,123]. For example, both Chromium (Cr) and Cuprum (Cu) noticeably inhibited naphthalene adsorption by biochar in aqueous solutions [124]. Chemical adsorption and ion exchange were the main adsorption mechanisms of biochar for heavy metals while physical adsorption was the mechanism for organic pollutants. The coexistence of heavy metals weakens the physical adsorption of organic pollutants by biochar [125]. However, there also exist opposing opinions. For example, the presence of a low concentration of Cu in solutions positively enhanced the adsorption of TC on biochar [126]. Cu can act as a bridge between biochar and TC, while the complexes of Cu and TC exhibited a stronger affinity for biochar [127]. Similar results were also reported when Cadmium (Cd) coexisted with sulfamethoxazole (SMX) [128]. Besides the “bridge” explanation of Cd, Cd adsorbed on biochar could mitigate the negative surface charge of biochar surface, then slow down the electrostatic repulsion between the biochar and the anion SMX [129]. The Cd-SMX complex has a higher affinity for biochar than SMX, thus accelerating SMX transfer efficiency to biochar [130]. In addition, Cd slows down the competition between SMX and water for adsorption sites by reducing the surrounding hydrophobicity [131].

## 4. Remediation Mechanisms of Emerging Pollutants by Biochar

Biochar serves as a powerful adsorbent for a range of emerging pollutants, thereby playing a crucial role in governing the fate, transport, and subsequent risk associated with pollutants [132,133,134,135]. The mechanisms for emerging pollutants removal by biochar will be discussed in detail in this section. Studies have shown that biochar’s sorption is controlled by the relative carbonized and non-carbonized fractions as well as their surface area and bulk properties [136,137]. For example, Zhu et al. [138] indicated that aromatic contaminants’ adsorption to wood chars was assisted by π-electron interactions, while proposing a pore-filling mechanism contributing to the sorption process for organic pollutants onto biochar. It has been also demonstrated that biochar can also serve as a catalyst for the degradation of some emerging pollutants. A recent study has found that biochar could not only be used as an adsorbent but also accelerate the reduction of nitrobenzene [139].

**Figure 2:**
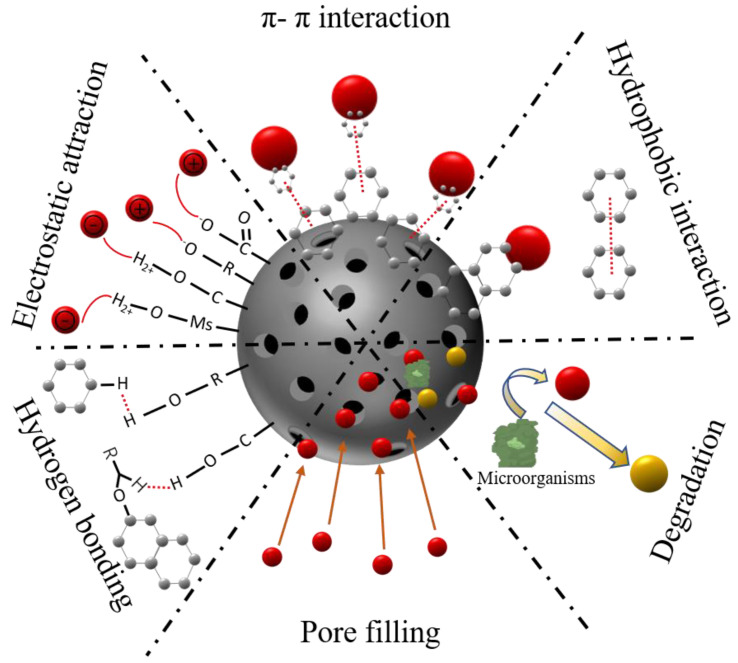
Possible mechanisms of biochar for emerging pollutants adsorption.

Usually, the organic pollutants adsorption process by biochar involves a combination of different interactions [140]. Figure 2 shows the conceptual illustration of organic pollutants’ adsorption mechanisms onto biochar, including (1) The π-π interactions between pollutants molecules and graphene layers of biochar; (2) the mechanisms of the direct electrostatic attraction/repulsion; (3) the inter-molecular hydrogen bonding; (4) hydrophobic interaction; (5) pore filling; (6) as a catalyst for the degradation of the pollutants (e.g., accelerates nitrobenzene reduction) [140].

The π-π EDA interactions typically occur in the sorption of aromatic compounds dominated by biochar on the graphene structure surface [141,142]. π-π EDA interactions are an important class of non-covalent interactions that contribute to the structure of biomolecules, chemical bonding, and the structure and properties of π-conjugated materials with aromatic rings, such as biochar [143]. During pyrolysis, irregular charge sharing between the aromatic rings of biochar leads to an increase or decrease in the electron density of biochar, forming a π-electron enriched or π-electron deficient medium [144]. When biochar is prepared below 500 °C, the π aromatic system acts as an electron acceptor because of the greater abundance of highly polar functional groups, while when pyrolyzed at temperatures above 500 °C, the biochar is used as an electron donor by binding escaped electron molecules [27,145,146]. Graphene-like structure in biochar expands with increasing pyrolysis temperature, thus increasing the π-π interaction [145]. It has been reported that pollutant molecules containing nitro groups can reduce the heterocyclic ring electron density and thus enhance the π-electron deficiency which resulted in stronger π-π interactions [147].

Electrostatic interaction is a vital system for the adsorption of ionizable and ionic organic chemicals on biochar. The pH largely determines the effect of electrostatic interaction in the adsorption process [148]. The interaction of biochar with pollutants at different pH conditions has been discussed in Section 3.3. In addition, the ionic strength has a great influence on the magnitude of the electrostatic force in the adsorption [148]. Organic pollutants with high electronegativity can be bonded to polar hydrogen bonds on the surface of biochar [149]. For example, the adsorption of dibutyl phthalate by modified sludge mass biochar is mainly through hydrogen bonding between the hydrogen atoms on the biochar and the oxygen atoms of the ester group on dibutyl phthalate [150].

The rise of pyrolysis temperature reduces the quantity of polar functional groups on the biochar surface, thus increasing its hydrophobicity. Its hydrophobic surface can adsorb non-polar organic molecules through hydrophobic interaction. Therefore, hydrophobic interaction is an important system for different hydrophobic organic compounds to adsorb on the biochar graphene-like surface [151]. Hydrophobic adsorption is accompanied by the partitioning mechanism of some organic molecules. In the partitioning process, the adsorbed organic compounds diffuse in the biochar pores or the organic matter in the noncarbonized part and then dissolve in the organic matter matrix [152]. Due to the competition between non-polar molecules and water molecules, the hydration energy required for hydrophobic interaction is usually lower than that for partition interaction [153].

The abundance of pores on biochar, as well as its huge specific surface area, determines the importance of the pore-filling mechanism in organic pollutant adsorption. Usually, organic compound molecules with small particle sizes are prone to be adsorbed through the pore-filling mechanism, the biochar pores are mainly micropores (<2 nm) and mesopores (2–50 nm) [134,154,155]; however, organic molecules with large pore size are difficult to be adsorbed by biochar because of size exclusion effect [97]. Studies have shown that the porous structure of the biochar surface has a dual effect on microbial degradation of organic pollutants: (1) organic pollutants enter the porous structure of biochar and cannot be directly used by microorganisms, reducing the degradation rate of organic pollutants by microorganisms, (2) porous structure is a good habitat for microorganisms such as algae, bacteria and fungi, improving microorganisms abundance and activity, and alter the microbial community structure under the pollutant stress [155].

In addition, the partially easily decomposed carbon sources and nitrogen sources on the biochar’s surface are beneficial to increase the abundance and activity of microorganisms. Meanwhile, the porous structure of biochar can store water and nutrients, becoming a microenvironment for microbes, and providing a shelter for the growth of special taxa of microorganisms, thus promoting the degradation of the organic pollutants [156,157,158].

## 5. Conclusions

Biochar, as an effective adsorbent, plays an important role in the management of emerging pollutants in aquatic systems. However, one type of biochar may not be appropriate for the removal of all types of emerging pollutants. Previous studies have shown promising results that biochar is an effective adsorbent and that the absorption performance of biochar is affected by parameters such as adsorbent dosage, adsorption time, adsorption temperature, types of pollutants and pH. Though biochar is effective in removing a variety of emerging pollutants from water, there are still some risks, problems and shortcomings for biochar in practical application and future research, which are listed as follows:

It can be concluded that the single production or modification method of biochar sometimes cannot meet the specific application conditions. In addition, some modification methods may reduce the stability of biochar in the environment, making it easier to be degraded, resulting in the release of endogenous pollutants from biochar. Therefore, multiple production or modification methods need to be explored in the future to solve complex environmental problems.

Although biochar can adsorb pollutants in aquatic systems, the pollutants are only transferred from the liquid phase to the adsorbent surface, without removal from the environment. Biochar application can lead to the accumulation of contaminant residues in the biochar itself. Whether these pollutants will release into the water bodies again is still unknown, and a question that needs to be addressed. In future research, it is necessary to systematically study the ecological and health risk effects of biochar in real applications, so as to serve the safe and effective use of biochar.

So far, studies mostly focus on the removal of a single emerging pollutant; however, the wastewater usually contains various emerging pollutants. More studies are needed to focus on sorption behavior in multi-solute systems.

## Data Availability

All the data have been included in the manuscript; others if any, are available from the corresponding author upon reasonable request.
